# 

**Published:** 1928

**Authors:** 


WE record with great sorrow the death of the Chancellor of the University of Bristol, LORD HALDANE, on August 19th, 1928. Our Chancellor was more than a titular head of the University: he took a keen and active interest in its progress and paid frequent visits to Bristol, his last being on the occasion of the Degree Congregation in June of this year. Of his eminence as a statesman and a lawyer this is not the place to speak. But a medical journal may at any rate express appreciation of him as a philosopher, and pay some meed of tribute to a great War Minister and patriot.

Mr. E. Watson-Williams showed cases of atresia and hyperostosis of the external ear and others illustrating results obtained by operative treatment of malignant disease of the larynx.

Dr. Kenneth Wills showed cases of rodent ulcer and other dermatological diseases.

Dr. A. D. Fraser showed pathological specimens.

---

### The Medical Library of the University of Bristol,

WITH WHICH IS INCORPORATED

**The Library of the Bristol Medico-Chirurgical Society.**

*The following donations have been received since the publication of the List in June,* 1928.

September, 1928.

| | | |
|---|---|---|
| *Liège Université Bibliothèque* (1) .. .. .. | 1 | volume |
| Michell Clarke Memorial Fund (2) .. .. | 1 | ,, |
| Dr. H. B. Murgoci (3) .. .. .. .. | 1 | ,, |
| New York University and Bellevue Hospital Medical College (4) .. .. .. .. | 1 | ,, |
| Mr. W. M. Ogilvie (5) .. .. .. .. | 1 | ,, |
| Rockefeller Foundation (6) .. .. .. | 1 | ,, |
| Royal College of Physicians of London (7) .. | 1 | ,, |
| Sheffield University, Medical Faculty (8) .. | 1 | ,, |
| Surgeon-General of the United States Army (9) | 1 | ,, |

Unbound periodicals have also been received from Professor E. W. Hey Groves and Dr. Carey Coombs.

### THE ONE HUNDRED AND TWENTY-FIFTH LIST OF BOOKS.

The figures in round brackets refer to the figures after the names of the donors. The books to which no such figures are attached have either been bought from the Library Fund or received through the *Journal.*

| | | | | |
|---|---|---|---|---|
| **Brend, W. A.** .. .. | *Handbook of Medical Jurisprudence* .. 6th Ed. | | 1928 |
| **Gravis, A.** .. .. .. .. | *Récherches anatomiques et physiologiques sur le tradescantia virginica L.* .. .. .. .. .. | (1) | 1898 |
| *Medical Department of the United States Army in the World War* | Vol. 9 | (9) | 1928 |
| *Notes on Clinical Laboratory Methods* .. .. .. .. .. .. .. .. .. .. | | (3) | 1927 |
| **Ogilvie, W. H.** .. .. | *Recent Advances in Surgery* .. .. .. .. .. | | (5) | 1928 |
| **Orrin, H. C.** .. .. | *Fascial Grafting in Principle and Practice* .. | | | 1928 |
| **Osler and McCrae** .. | *Modern Medicine.* Vol. 6 .. .. 3rd Ed. | (2) | 1928 |
| **Porter, W. S.** .. .. | *The Medical School in Sheffield,* 1828-1928 | (8) | 1928 |
| *Tercentenary Celebration of the Publication of the "De Motu Cordis" by William Harvey,* 1628. | *Programme of Arrangements* .. .. .. .. .. | (7) | 1928 |
| **Todd, A. T.**.. .. .. | *Chemotherapeutic Researches on Cancer* .. .. | | 1928 |

TRANSACTIONS, REPORTS, JOURNALS, ETC.

*Collected Reprints from the Department of Experimental Surgery, New York University and Bellevue Hospital Medical College.* Vol. 5 .. (4) 1926-27
*Methods and Problems of Medical Education.* 9th Series .. .. .. (6) 1928
*Progressive Medicine,* 1928. Vols. 1 and 2 .. .. .. .. .. .. .. .. .. 1928
*Surgical Clinics of North America.* Vol. 8. No. 3 .. .. .. .. .. .. 1928

### Local Medical Notes.

Dr. MacDonald Critchley has been appointed Assistant-Physician to the National Hospital, Queen Square.

EXAMINATION RESULTS.

**University of Bristol.**—Students of the University have recently passed the following examinations :—

M.B., CH.B.—*First Examination. Pass :* S. B. Adams, A. J. Board, G. M. Evans, Miss M. A. W. Hawkes, F. J. W. Lewis, R. L. Marks, H. E. Pearse, Miss F. E. Powell, S. L. Prescott, O. E. L. Sampson, J. P. P. Stock.

M.B., CH.B.—*Second Examination.* (*Part I.*). *Pass :* G. M. Evans, F. E. Fletcher, R. L. Marks, J. P. P. Stock. *In Organic Chemistry* (*Completing Examination*) *:* J. Ridgway. *In Elementary Anatomy* (*Completing Examination*) *:* G. T. Bevir, W. R. Cambridge. *In Elementary Anatomy only :* H. James.

M.B., CH.B.—*Second Examination.* (*Part II.*). *Pass :* J. S. Adamson, J. E. Brown, C. J. N. Davis, L. C. Holland, C. N. Royal.

FINAL M.B., CH.B. (*Part II.*).—*Completing Examination. Pass with 2nd Class Honours :* T. H. Berrill (with distinction in Special Pathology). *Pass :* B. J. Boulton, R. D. Jenkins (with distinction in Public Health), Miss H. B. Murgoci (with distinction in Special Pathology), T. B. Wansbrough (with distinction in Obstetrics). *In Group II.* (*Completing Examination*) *:* D. E. C. Andrew, A. J. McD. Grimston.

B.D.S.—*Second Examination. Pass in Physiology and Anatomy* (*Completing Examination*) *:* M. J. Ryan.

FINAL B.D.S.—*Pass :* W. M. Evans.

L.D.S. (*Preliminary Science*).—*Pass : In Biology only* (*Completing Examination*) *:* Miss H. C. L. Blinkworth, R. E. W. Smith.

L.D.S.—*First Professional Examination. In Physiology and Anatomy* (*Completing Examination*). *Pass :* E. V. O'Hara, H. L. Saunders. *In Physiology and Anatomy only. Pass :* A. MacDonald Watson.

L.D.S.—*Second Professional Examination. In Dental Mechanics and Metallurgy* (*Completing Examination*). *Pass :* A. N. Moon, T. A. Satchwell, C. G. Westlake. *In Materia Medica* (*Completing Examination*). *Pass :* Miss B. J. Shapland. *In Dental Metallurgy and Mechanics only. Pass :* J. D. Galloway. *In Materia Medica only. Pass :* E. R. Carpenter. *In Dental Metallurgy and Materia Medica only. Pass :* I. W. Williams.

L.D.S.—*Final Professional Examination. Pass :* J. H. Edwards, H. J. Phillips, Miss S. M. Weir.

CONJOINT BOARD.—M.R.C.S., L.R.C.P. — *Preliminary Medical :* R. H. Moodie, C. A. G. Evans.

M.R.C.S., L.R.C.P.—*First Examination. Anatomy and Physiology :* D. P. Dewe. *Physiology :* Miss A. M. B. Walker. *Pharmacy and Materia Medica :* C. H. Pauli.

M.R.C.S., L.R.C.P.—*Final Examination :* H. D. Pyke (*Medicine and Surgery only*) ; A. C. Fisher (*Medicine, Surgery and Midwifery, Completing Examination*) ; L. E. Vine (*Medicine, Surgery and Midwifery, Completing Examination*) ; L. H. Wharton (*Midwifery only*) ; D. R. House (*Surgery and Midwifery, Completing Examination*) ; T. H. Berrill (*Surgery and Midwifery, Completing Examination*) ; Miss C. F. Fox (*Surgery, Completing Examination*).

M.R.C.S., L.R.C.P.—*Diploma in Tropical Medicine and Hygiene :* E. R. Wide (M.B., Ch.B., Bristol).

L.D.S., R.C.S.—*Final Examination* (*Part I.*) *:* H. J. Phillips, R. G. Boodle, C. Haskins.

L.D.S., R.C.S.—*Final Examination* (*Part II.*) *:* H. J. Phillips, R. A. Phillips.

SOCIETY OF APOTHECARIES.—L.S.A.—*Forensic Medicine :* T. A. Barnabas.

L.S.A.—*Midwifery :* T. A. Barnabas, N. H. Kettlewell, L. P. Gregory.

L.S.A.—*Medicine :* T. A. Barnabas.

### Publications Received.

From J. W. ARROWSMITH LTD.

*Chemotherapeutic Researches on Cancer.* By A. T. TODD, M.B. (Edin.), M.R.C.P. (Lond.). 1928. Price 2s. 6d.

From J. & A. CHURCHILL.

*A Code of Rules for the Prevention of Communicable Diseases in Schools.* Issued by the Medical Officers of Schools Association. Ninth Edition. 1928. Price 2s. 6d. net.

From WILLIAM HEINEMANN, LTD.

*Low Blood Pressure.* By J. F. HALLS DALLY, M.A., M.D., B.Chir. (Cantab.), M.R.C.P. (Lond.). 1928. Price 15s. net.

*The Radiography of the Chest.* By W. OVEREND, M.A., M.D. (Oxon.), B.Sc. (Lond.). Vol. II. 1928. Price 21s. net.

*The Tonsils and Adenoids and their Diseases.* By I. MOORE, M.B., C.M. (Edin.). 1928. Price 21s. net.

*Dental Medicine.* By F. W. BRODERICK, M.R.C.S., L.R.C.P., L.D.S. (Eng.). 1928. Price 21s. net.

From THE LANCET, LTD.

*Guy's Hospital Reports.* Vol. 78. No. 2. April, 1928. 12s. 6d. net. Annual subscription 42s. net.

*Guy's Hospital Reports.* Vol. 78. No. 3. July, 1928. Price 12s. 6d. net. Annual subscription 42s. net.

From H. K. LEWIS & CO. LTD.

*Deafness and its Alleviation.* By V. NESFIELD, F.R.C.S. 1928. 7s. 6d. net.

*Extra Pharmacopoeia.* By MARTINDALE & WESTCOTT. Vol. 1. Nineteenth Edition. 1928. Price 27s. 6d. net.

*A Shorter Anatomy.* By E. WOLFF, M.B., B.S. (Lond.), F.R.C.S. (Eng.). 1928. Price 18s. net.

*Handbook of Sanitary Law.* By B. B. HAM, M.D., D.P.H. (Camb.). Tenth Edition. 1928. Price 6s. 6d. net.

*Insomnia and Drug Addiction.* By P. C. COLLINGWOOD FENWICK. 1928. Price 2s. net.

*A Pocket Medical Dictionary.* By GEORGE M. GOULD, A.M., M.D. 1928. Price 10s. net.

From OLIVER & BOYD.

*Fascial Grafting in Principle and Practice.* By H. C. ORRIN, O.B.E., F.R.C.S. (Edin.). 1928. Price 7s. 6d. net.

From OXFORD UNIVERSITY PRESS.

*Gastro-intestinal Diseases.* Lectures delivered at the James McKenzie Institute for Clinical Research, St. Andrews. 1927. Edited by D. WATERSTON, M.A., M.D., F.R.C.S. (Edin.). 1928. Price 10s. 6d. net.

*The Robert Jones Birthday Volume: A Collection of Surgical Essays.* 1928. Price 42s. net.

*Gynæcology with Obstetrics.* By JOHN S. FAIRBAIRN, M.A., B.M., B.Ch., F.R.C.P., F.R.C.S. Second Edition. 1928. Price 25s. net.

From JOHN WRIGHT & SONS LTD.

*Some Principles of Diagnosis, Prognosis and Treatment.* By ROBERT HUTCHISON, M.D., F.R.C.P. 1928. Price 2s. 6d. net.

### Bristol Medico-Chirurgical Journal.

Published Quarterly (royal 8vo reduced) under the auspices of the Bristol Medico-Chirurgical Society.

*An excellent means of advertising*

The only Medical Journal published in the West of England, and has an extensive circulation not only amongst the Medical men of Bristol, but also amongst those of the adjoining Counties and South Wales, and in exchange goes to all parts of the world.

Terms for Advertisements:

Whole Page, £2 2s., Half Page, £1 5s., Quarter Page, 15s. per issue.

***PRICES FOR SPECIAL POSITIONS ON APPLICATION.***

J. W. ARROWSMITH LTD.,

Advertising Agents,

11 Quay Street, BRISTOL.

